# Why do on-pump patients live longer?

**DOI:** 10.18632/aging.101607

**Published:** 2018-10-18

**Authors:** Nicola King, Val Mann

**Affiliations:** 1 School of Biomedical Sciences, Faculty of Medicine and Dentistry, University of Plymouth, Plymouth PL4 8AA, UK

**Keywords:** coronary artery bypass grafting, cardiopulmonary bypass, off pump, mortality, cognitive decline

For the past 3 decades there has been a heated controversy in the world of coronary artery bypass graft (CABG) surgery. This has concerned whether to perform CABG with (on pump) or without (off pump) cardiopulmonary bypass. Each technique has advantages and shortcomings. For example on pump has often been associated with a small risk of stroke, whereas off pump reduces aortic manipulation [1]. Both approaches have been implicated in a systemic inflammatory response [1]. Despite >60 meta-analyses investigating this topic no conclusive answer has been reached and the debate continues [1].

Two of the problems encountered when seeking evidence are the sparsity of large randomised controlled trials (RCTs) and a focus on short-term clinical outcomes. A possible resolution to the latter was the long awaited publication of the five-year outcomes of two of the largest RCTs to date, ROOBY (Randomized on/off bypass) [2] and CORONARY (CABG off or on pump revascularization) [3] trials. This enabled a fresh meta-analysis investigating long-term clinical outcomes (mortality, myocardial infarction, stroke, angina and the need for revascularisation) incorporating 6 RCTs, and, most importantly, including ROOBY and CORONARY. The results showed a small but significant benefit of on pump in terms of mortality with all other comparisons showing no differences [4]. This raises a new question what is the reason/mechanism underlying on pump’s seemingly superior long-term survival rate?

A criticism that is often levelled at off pump CABG is the under achievement of complete revascularisation. This might arise from the greater technical challenge of performing distal anastomoses on the beating heart, although surgeon experience is thought to be a factor [1]. In the CORONARY trial significantly fewer grafts were placed in the off pump group, and the rate of incomplete revascularisation was higher in the off pump group, though the p value was borderline significant (p=0.05) [5]. A similar pattern was observed in ROOBY where less grafts were placed in the off pump group and the number of grafts placed that was lower than planned was significantly higher in the off pump group [6]. The consequences of this might be a significant re-emergence of angina, higher occurrence of myocardial infarction and a greater need for revascularisation in the off pump group. Only 3 of the 6 RCTs in the long-term comparison meta-analysis reported the incidence of angina at >4 years including CORONARY but not ROOBY [4]. Yet the result was insignificant [4]. Both CORONARY and ROOBY reported the incidence of repeat revascularisation (plus three other studies) yielding an insignificant odds ratio of 1.15 (95% confidence interval [CI] 0.95 to 1.40) overall. The same five RCTs reported the incidence of myocardial infarction and again the odds ratio (1.06 [95% CI 0.91 to 1.25]) for the comparison was insignificant [4]. It is noteworthy that in the 5-year follow ups published about the CORONARY and ROOBY RCTs death from cardiovascular causes was measured. No significant difference was found for off pump compared to on pump [2,3].

If greater susceptibility to cardiovascular events including stroke is not the underlying mechanism what is the cause of the lower longevity in the off pump group? Another parameter that was measured in CORONARY that could be a cause of premature death was new renal failure requiring dialysis. However at five years there was no difference in the rate in the off pump group compared to on the pump group [3]. It is well known that as time passes patients undergoing CABG are becoming older and have a higher number of comorbidities. There is also a body of evidence to suggest that off pump is favoured in high risk patients [1]. Equally well known is that advancing age and lifestyle choices are risk factors for the development of dementia. Could there be a difference in the susceptibility of the off and on pump groups to the onset and progression of dementia? An early warning sign of the onset of dementia is cognitive decline. This was measured by Van Dijk et al in their five year follow-up to an RCT comparing off versus on pump. A reduction in cognitive performance occurred in both groups; however, it only reached significance in the on pump group [7]. Another dead end lies in comparing health related quality of life, which was similar for surviving patients at follow-up for 3 RCTs [5,7,8].

In conclusion a recent meta-analysis found a small but significant long-term survival advantage for patients undergoing on pump CABG compared to those undergoing off pump CABG. Several avenues including cardiovascular causes, renal causes, dementia causes and health related quality of life causes have been explored in the search for an underlying mechanism to explain on pump’s long-term superiority with respect to mortality. Unfortunately, no satisfactory explanation has been reached and the question as to why on pump is better in terms of mortality in the long-term requires further investigation.

## References

[1] Gaudino M, et al. J Am Heart Assoc. 2018; 7:e009934. https://www.ahajournals.org/doi/10.1161/JAHA.118.009934 30369328 10.1161/JAHA.118.009934 PMC6201399

[2] Shroyer AL, et al. N Engl J Med. 2017; 377:623–32. 10.1056/NEJMoa1614341 28813218

[3] Lamy A, et al. N Engl J Med. 2016; 375:2359–68. 10.1056/NEJMoa1601564 27771985

[4] Smart NA, et al. J Am Coll Cardiol. 2018; 71:983–91. 10.1016/j.jacc.2017.12.049 29495998

[5] Lamy A, et al. N Engl J Med. 2012; 366:1489–97. 10.1056/NEJMoa1200388 22449296

[6] Shroyer AL, et al. N Engl J Med. 2009; 361:1827–37. 10.1056/NEJMoa0902905 19890125

[7] van Dijk D, et al. JAMA. 2007; 297:701–08. 10.1001/jama.297.7.701 17312289

[8] Angelini GD, et al. J Thorac Cardiovasc Surg. 2009; 137:295–303. 10.1016/j.jtcvs.2008.09.046 19185140 PMC2836483

